# Mirogabalin Decreases Pain-like Behaviours and Improves Opioid and Ketamine Antinociception in a Mouse Model of Neuropathic Pain

**DOI:** 10.3390/ph15010088

**Published:** 2022-01-13

**Authors:** Renata Zajączkowska, Ewelina Rojewska, Agata Ciechanowska, Katarzyna Pawlik, Katarzyna Ciapała, Magdalena Kocot-Kępska, Wioletta Makuch, Jerzy Wordliczek, Joanna Mika

**Affiliations:** 1 Department of Interdisciplinary Intensive Care, Jagiellonian University Medical College, 30-688 Krakow, Poland; j.wordliczek@uj.edu.pl; 2 Department of Pain Pharmacology, Maj Institute of Pharmacology Polish Academy of Sciences, 31-343 Krakow, Poland; rojewska@if-pan.krakow.pl (E.R.); ciechan@if-pan.krakow.pl (A.C.); pawlik@if-pan.krakow.pl (K.P.); kciapala@if-pan.krakow.pl (K.C.); makuch@if-pan.krakow.pl (W.M.); 3 Department of Pain Research and Treatment, Jagiellonian University Medical College, 31-501 Krakow, Poland; magdalena.kocot-kepska@uj.edu.pl

**Keywords:** mirogabalin, neuropathic pain, morphine, buprenorphine, oxycodone, ketamine

## Abstract

Neuropathic pain remains a difficult clinical challenge due to its diverse aetiology and complex pathomechanisms, which are yet to be fully understood. Despite the variety of available therapies, many patients suffer from ineffective pain relief; hence, the search for more efficacious treatments continues. The new gabapentinoid, mirogabalin has recently been approved for clinical use. Although its main mechanism of action occurs at the α2σ-1 and α2σ-2 subunits of calcium channels and is well documented, how the drug affects the disturbed neuropathic interactions at the spinal cord level has not been clarified, which is crucial information from a clinical perspective. The findings of our study suggest that several indirect mechanisms may be responsible for the beneficial analgesic effect of mirogabalin. This is the first study to report that mirogabalin enhances the mRNA expression of spinal antinociceptive factors, such as IL-10 and IL-18BP, and reduces the concentration of the pronociceptive substance P. Importantly, mirogabalin improves the morphine-, buprenorphine-, oxycodone-, and ketamine-induced antinociceptive effects in a neuropathic pain model. Our findings support the hypothesis that enhancing opioid and ketamine analgesia by combining these drugs with mirogabalin may represent a new strategy for the effective pharmacotherapy of neuropathic pain.

## 1. Introduction

**Scheme 1:**
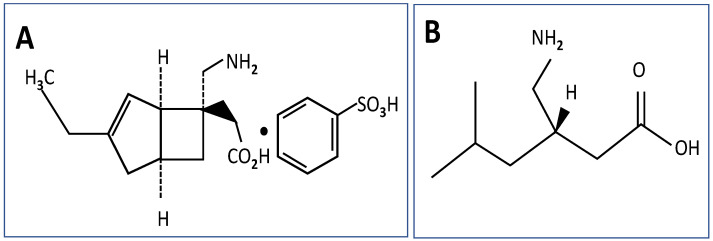
Chemical structures: mirogabalin—(**A**), pregabalin—(**B**).

The treatment of neuropathic pain remains a tremendous challenge due to its diverse aetiology and complex pathomechanisms, which are still not fully understood. Despite the various therapies available, many patients suffer from ineffective pain relief. Epidemiological studies show that only half of patients can achieve 30–50% pain relief, while the remainder cannot unfortunately be helped [1]. Therefore, the search for new and more effective treatments for neuropathic pain continues. First-line drugs in the treatment of this kind of pain include antidepressants (tricyclic antidepressants and serotonin-norepinephrine reuptake inhibitors) and gabapentinoids (gabapentin, pregabalin) [1]. A new gabapentinoid recently introduced into clinical practice is mirogabalin (C_12_H_19_NO_2_, molar mass 209.289 g/mol, Scheme 1A). In January 2019, mirogabalin was registered in Japan for the treatment of peripheral neuropathic pain following promising clinical trials in patients with postherpetic neuralgia and painful diabetic polyneuropathy [2]. Compared with pregabalin (C_8_H_17_NO_2_, molar mass 159.23 g/mol, Scheme 1B) mirogabalin has a higher affinity for the α2δ-1 and α2δ-2 subunits of calcium channels and dissociates more slowly from the α2δ-1 subunit than from the α2δ-2 subunit, which results in its superior efficacy and lower incidence of adverse effects. After single or repeated doses, mirogabalin is quickly absorbed, and the average time to maximum plasma concentration (Tmax) is 0.5–1.5 h. The steady-state plasma concentration is achieved by day 3. After oral administration, mirogabalin is quickly converted into its free form, in which A200-700 is the main active circulating isoform. Ninety-nine percent of mirogabalin is eliminated through the kidneys, with only 1% of the dose excreted in the faeces [3].

Though the main mechanism of action of mirogabalin is well known, it is still unclear how mirogabalin effects neuroimmune interactions which are disrupted in neuropathy at the spinal cord level. This is extremely important from a clinical perspective. Currently, pain is considered as a neuro-immune disorder, since it is known that the activation of immune and immune-like glial cells results in the release of both pro- and anti-nociceptive factors.

Due to the difficulties in obtaining effective pain relief, in the treatment of neuropathic pain, combination therapy is frequently used [4]. One of the therapeutic options consists of administering gabapentinoids in combination with opioids [5,6]. The Cochrane Database of Systematic Reviews [4] shows that gabapentin administered with morphine [7] or oxycodone [8] has a better analgesic effect than gabapentin alone. Clinical studies have also shown that the use of low-dose ketamine infusion as an adjuvant to oral gabapentin produces a better analgesic effect in patients who developed neuropathic pain following spinal cord injury than gabapentin alone. Interestingly, the authors also found that the analgesic effect of ketamine infusion was maintained for 2 weeks after the infusion was discontinued [9]. To the best of our knowledge, experimental and clinical studies have not assessed the efficacy of the combined use of the new gabapentinoid mirogabalin with opioids and ketamine.

Therefore, we first investigated the effect of a single intraperitoneal (*i.p.*) administration of mirogabalin on already fully developed tactile and thermal hypersensitivity in mice, following chronic constriction injury (CCI) of the sciatic nerve (Bennett model). Second, we studied how a single *i.p.* mirogabalin administration influences opioid (morphine, buprenorphine and oxycodone) and ketamine antinociception in the neuropathic pain model. Our third goal was to assess the effect of pre-emptive and repeated (for 7 days) *i.p*. mirogabalin injection on the development of pain-related behaviour following CCI, and its concurrent impact on the mRNA of cellular markers, such as *Iba-1, Tmem119, Gfap, Cd4* and *Cd8*, and spinal changes in the expression of pronociceptive (*IL-1beta, IL-6, IL-18)* and antinociceptive (*IL1-alpha, IL-10, IL-18BP*) mRNA factors, including certain neuropeptides involved in nociceptive transmission (*substance P (SP), neuropeptide Y (NPY),* and *calcitonin gene-related peptide (CGRP)*). We also examined the impact of the abovementioned procedure on opioid prohormones [*proopiomelanocortin (Pomc), proenkephalin (Penk), prodynorphin (Pdyn),* and *pronocicepitin (Pnoc)*] and opioid receptors [*mu (Mor), delta (Dor), kappa (Kor)* and *nociceptin (Nor*)].

## 2. Results

### 2.1. Effects of a Single i.p. Mirogabalin Administration on Pain-Related Behaviour Measured 11 Days after CCI in Mice

**Figure 1:**
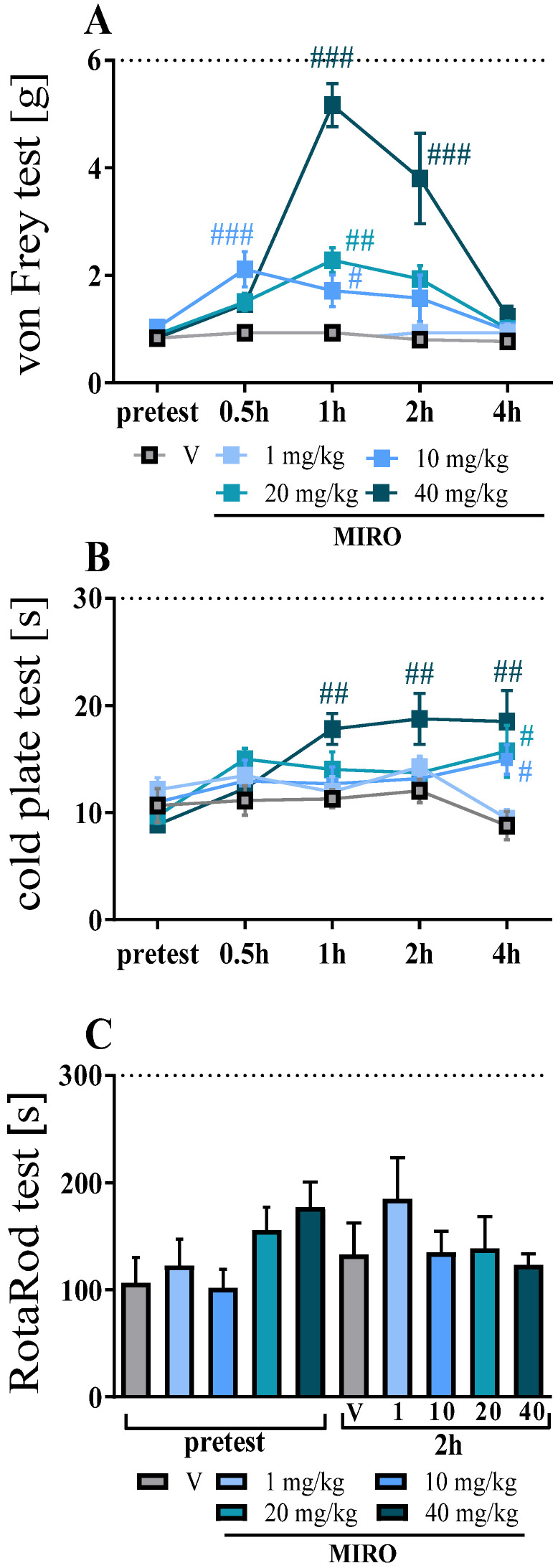
Effect of single intraperitoneal (*i.p*.) administration of mirogabalin (1, 10, 20, 40 mg/kg) on pain-related behaviours in mice on day 11 post-CCI (von Frey test—**A**; cold plate test—**B**; rotarod test—**C**). The horizontal dotted line represents the cut-off value. The data are presented as the means ± SEM (6–7 mice per group). Intergroup differences were analysed based on one-way ANOVA with Bonferroni’s multiple comparisons post hoc test. # *p* < 0.05, ## *p* < 0.01, ### *p* < 0.001 indicate a significant difference between V-treated and mirogabalin-treated CCI-exposed animals. Additionally, the results were evaluated using two-way ANOVA to determine the time × drug interaction (please see results in Section 2.1). Abbreviations: MIRO—mirogabalin; V—vehicle.

A single *i.p.* administration of mirogabalin at doses of 1, 10, 20 and 40 mg/kg was administered 11 days after CCI, i.e., after hypersensitivity had been fully established. The effect of mirogabalin administration on the development of hypersensitivity to tactile and thermal stimuli was measured using von Frey (Figure 1A) and cold plate (Figure 1B) tests, respectively, at 0.5, 1, 2, and 4 h after drug administration. One-way ANOVA have shown the greatest analgesic effect after mirogabalin administration at a dose of 40 mg/kg, which significantly (*p* < 0.0001) attenuated tactile hypersensitivity 1 h after injection in the von Frey test (Figure 1A). This analgesic effect was observed until 2 h after injection [F_(4, 26)_ = 7.009, *p* = 0.0006] (Figure 1A). Moreover, the two-way ANOVA confirmed significant interaction [F_(4, 50)_ = 17.52; *p* < 0.0001] between the investigated treatment and the time points subjected to investigation. A single *i.p.* dose of mirogabalin (10 and 20 mg/kg) reduced hypersensitivity to tactile stimuli for only 1 h after injection. However, importantly, two-way ANOVA confirmed a significant interaction between the investigated treatment and the investigated time points for 20 mg/kg dose [F_(4, 50)_ = 6.604; *p* = 0.0002], but not for 10 mg/kg dose [F_(4, 55_ = 1.622; *p* = 0.1818]. The lowest dose of 1 mg/kg did not show any antinociceptive effect, as measured by one-way and two-way ANOVA (Figure 1A).

In the cold plate test, the pain perception threshold was elevated after the highest dose of mirogabalin (Figure 1B). One-way ANOVA showed effect after the administration of the highest dose of mirogabalin (40 mg/kg) from 1 h [F_(4, 26)_ = 3.194, *p* = 0.0292] to 4 h [F_(4, 26)_ = 4.787, *p* = 0.0050] (Figure 1B). Two-way ANOVA confirmed significant interaction [F_(4, 48)_ = 4.159; *p* = 0.0057] between the investigated treatment and the time points subjected to investigation. One-way ANOVA showed, in the cold plate test, that *i.p.* administration of mirogabalin (20 mg/kg) significantly reduced thermal hypersensitivity [F_(4, 26)_ = 4.787, *p* = 0.0050] 4 h after injection, compared with the vehicle treatment (Figure 1B). Similarly, the dose of 10 mg/kg attenuated thermal hypersensitivity [F_(4, 26)_ = 4.787, *p* = 0.0050] for 4 h after injection. In this case, two-way ANOVA did not show time × drug interaction [F_(4, 48)_ = 2.034; *p* = 0.1045] and [F_(4, 53)_ = 1.43; *p* = 0.2362] for 20 and 10 mg/kg doses, respectively. An injection of the lowest mirogabalin dose of 1 mg/kg did not show any antinociceptive effect in the cold plate test, as measured by one-way and two-way ANOVA (Figure 1B). Furthermore, the locomotor activity of mice was not altered. For all mirogabalin-treated groups, the rotarod performance test (1–40 mg/kg) exhibited no significant lengthening of riding time compared with the control (naïve) group (Figure 1C).

### 2.2. Effects of Repeated i.p. Mirogabalin Administration on Pain-Related Behaviour Measured 7 Days after CCI in Mice

**Figure 2:**
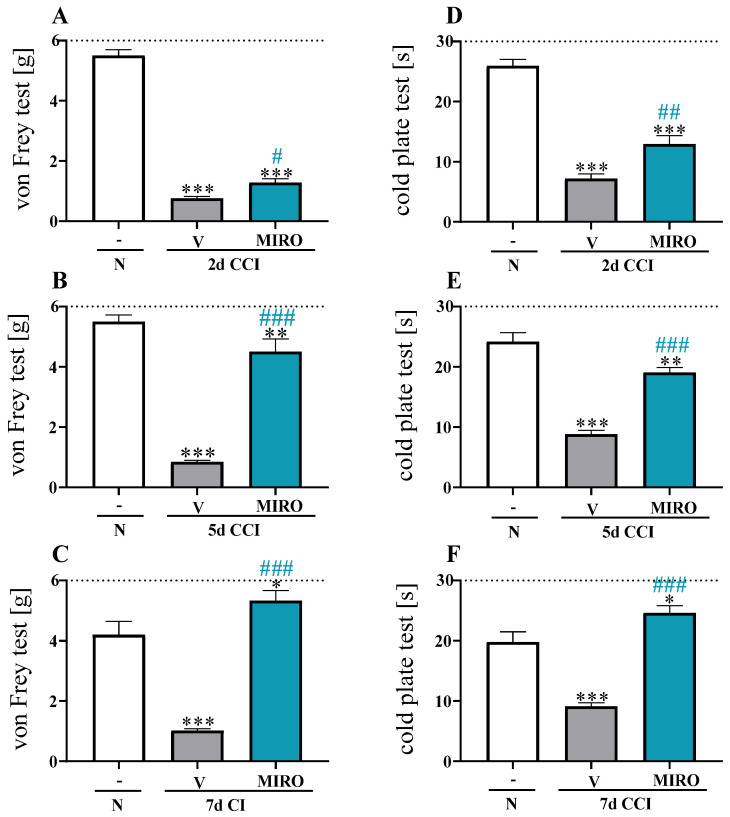
Effect of repeated intraperitoneal (*i.p.*) administration of mirogabalin (20 mg/kg) 16 h and 1 h before CCI and then twice daily for 7 days on tactile (von Frey-**A**–**C**) and thermal hypersensitivity (cold plate-**D**–**F**) in CCI-exposed mice as measured on days 2, 5 and 7 after chronic constriction injury. The horizontal dotted line represents the cut-off value. The data are presented as the means ± SEM (6–10 mice per group). Intergroup differences were analysed based on one-way ANOVA with Bonferroni’s multiple comparisons post hoc test. * *p* < 0.05, ** *p* < 0.01, *** *p* < 0.001 indicate differences between the naïve and V- or mirogabalin-treated CCI-exposed mice; # *p* < 0.05, ## *p* < 0.01, ### *p* < 0.001 indicates differences between V- and mirogabalin-treated CCI-exposed mice. Abbreviations: N—naïve; V—vehicle; MIRO—mirogabalin.

All the CCI-exposed mice developed tactile (*p* < 0.0001; Figure 2A–C) and thermal (*p* < 0.0001; Figure 2D–F) hypersensitivity relative to the control (naïve) group 2, 5 and 7 days following CCI. Mirogabalin was injected 16 h and 1 h before CCI and then twice per day for 7 days. The greatest analgesic effect of repeated *i.p.* administration of mirogabalin was observed 7 days post-CCI in the von Frey [F_(2,23)_ = 46.35, *p* < 0.0001] and cold plate [F_(2 23)_ = 36.14, *p* < 0.0001] tests, respectively (Figure 2C,F).

### 2.3. Effects of a Single i.p. Administration of Mirogabalin on the Efficacy of Morphine, Buprenorphine, Oxycodone and Ketamine Antinociception 7 Days after CCI in Mice

**Figure 3:**
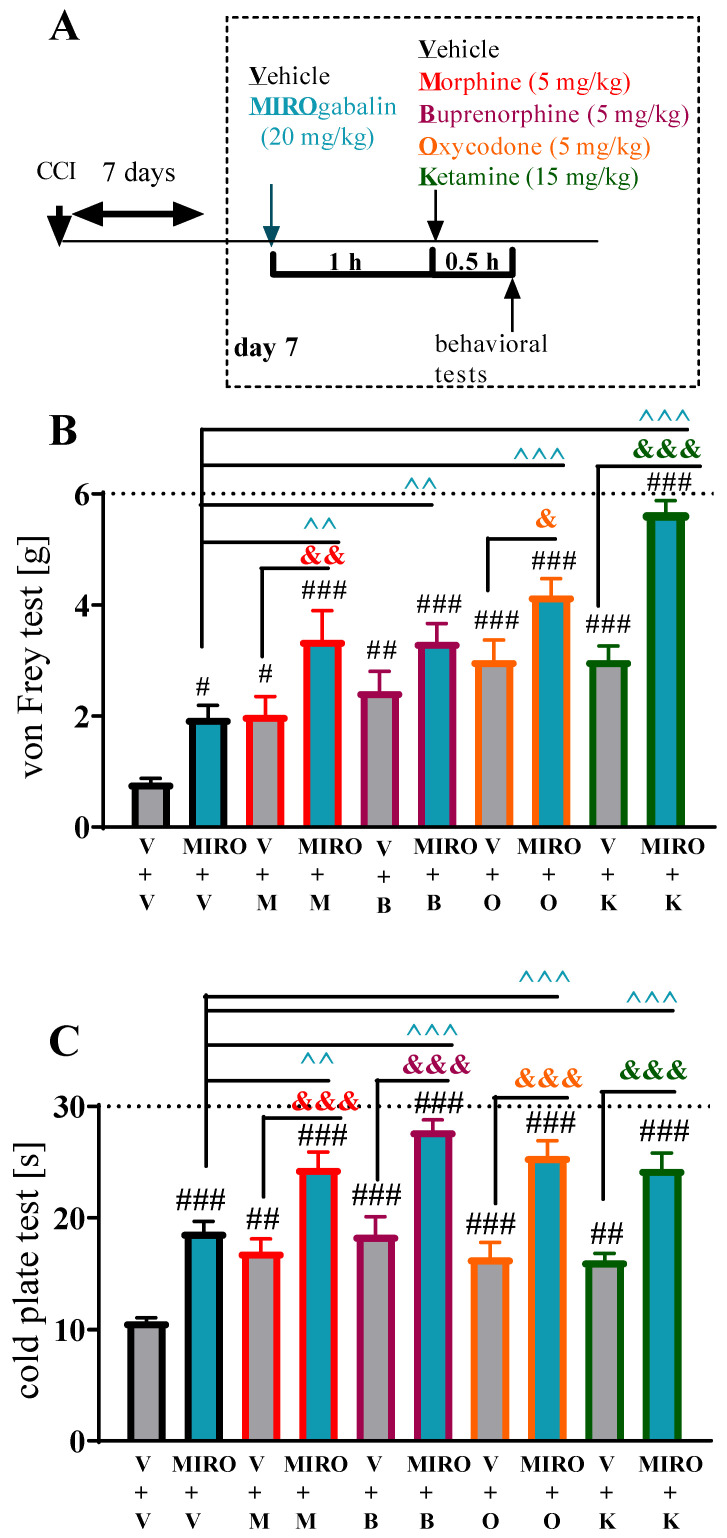
Effect of a single intraperitoneal (*i.p.*) administration of mirogabalin (20 mg/kg) on morphine (M; 5 mg/kg), buprenorphine (B; 5 mg/kg, oxycodone (O; 5 mg/kg), and ketamine (K; 15 mg/kg), effectiveness on day 7 after CCI (**A**) measured by tactile (**B**, von Frey test) and thermal (**C**, cold plate test) hypersensitivity. The data are presented as means ± S.E.M. (4–6 mice per group). The results were analysed using one-way ANOVA with Bonferroni’s post hoc multiple comparisons test. # *p* < 0.05, ## *p* < 0.01, ### *p* < 0.001 indicate difference compared with V-treated CCI-exposed mice; & *p* < 0.05, && *p* < 0.01, &&& *p* < 0.001 indicate differences between the V+M vs. MIRO+M, V+B vs. MIRO+B, V+O vs. MIRO+O and V+K vs. MIRO+K-treated CCI-exposed mice.; ^^ *p* < 0.01, ^^^ *p* < 0.001 indicate differences between MIRO+V vs. MIRO+M, MIRO+B, MIRO+O and MIRO+K-treated CCI-exposed mice. Abbreviations: CCI—chronic constriction injury; M—morphine; B—buprenorphine; O—oxycodone; K—ketamine; V—vehicle; MIRO—mirogabalin.

Tactile and thermal hypersensitivity were assessed using von Frey (Figure 3B) and cold plate (Figure 3C) tests, respectively, 0.5 h after the *i.p.* administrations of single morphine, buprenorphine, oxycodone (5 mg/kg each) and ketamine (15 mg/kg), so 1.5 h after mirogabalin (20 mg/kg) or Vehicle injections (Figure 3A). One-way ANOVA analysis revealed significant differences for von Frey (F_(9,48)_ = 14.78, *p* < 0.0001) and cold plate (F_(9,48)_ = 17. 73, *p* < 0.0001) tests. A single mirogabalin injection produced a significant antinociceptive effect compared with vehicle treatment in the von Frey [*p* < 0.05] and cold plate [*p* < 0.001] tests, respectively (Figure 3B,C). Similarly, the injections of morphine, buprenorphine, oxycodone or ketamine attenuated neuropathic pain symptoms in mice in both tests (Figure 3B,C). 

The combined administration of mirogabalin and morphine resulted in substantially more effective antinociception, following tactile [*p* < 0.01 vs. morphine; *p* < 0.01 vs. mirogabalin] and thermal [*p* < 0.001 vs. morphine; *p* < 0.01 vs. mirogabalin] hypersensitivity (Figure 3B,C). In the von Frey test, the administration of buprenorphine with mirogabalin revealed a reduction in tactile hypersensitivity; however, no significant differences were observed compared with the opioid administrated alone (Figure 3B). Nevertheless, a single *i.p.* mirogabalin and buprenorphine administration produces improved antinociception, as shown in the cold plate test [*p* < 0.001 vs. buprenorphine; *p* < 0.001 vs. mirogabalin] (Figure 3C). Moreover, mirogabalin and oxycodone co-administration evoked better antinociception in von Frey [*p* < 0.05 vs. oxycodone; *p* < 0.001 vs. mirogabalin] and cold plate [*p* < 0.001 vs. oxycodone; *p* < 0.001 vs. mirogabalin] tests (Figure 3B,C).

Importantly, injection of mirogabalin combined with ketamine also resulted in substantially more effective reduction of tactile [*p* < 0.001 vs. ketamine; *p* < 0.001 vs. mirogabalin] and thermal [*p* < 0.001 vs. ketamine; *p* < 0.001 vs. mirogabalin] hypersensitivity (Figure 3B,C).

### 2.4. Effects of Repeated i.p. Administration of Mirogabalin on the mRNA Levels of the Cellular Markers Iba-1, Tmem119, Gfap, Cd4 and Cd8 Measured on Day 7 after CCI in the Spinal Cord of Mice

**Figure 4:**
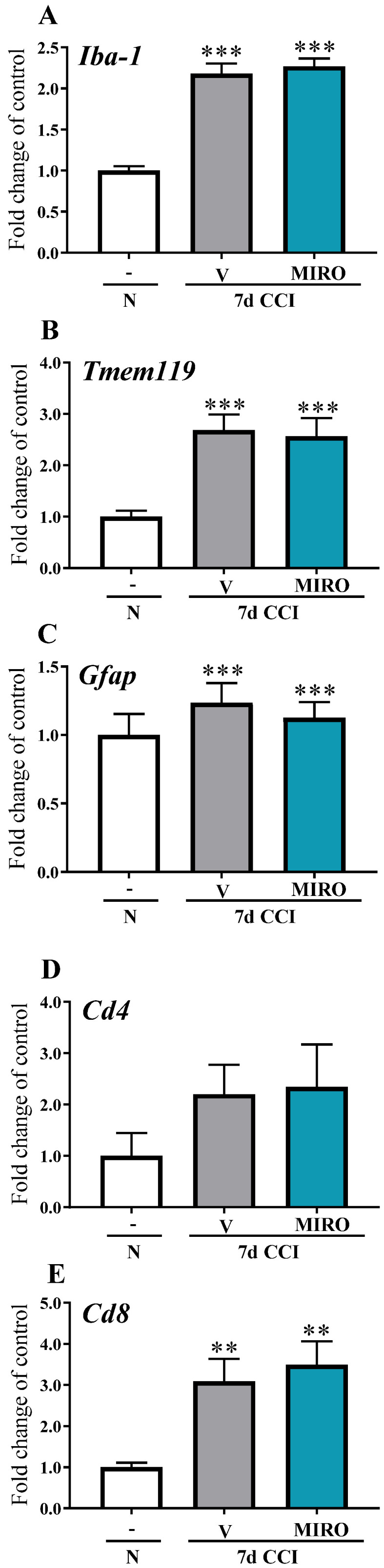
Effect of repeated intraperitoneal (*i.p.*) administration of mirogabalin (MIRO; 20 mg/kg) on *Iba-1* (**A**), *Tmem119* (**B**), *Gfap* (**C**) *Cd4* (**D**) and *Cd8* (**E**) mRNA levels in the spinal cord on day 7 post-CCI in mice. The data for the biochemical studies were analysed as fold changes compared with the controls and represent the normalized averages derived from the RT-qPCR threshold cycles of 5–10 samples from each group. Intergroup differences were analysed based on one-way ANOVA with Bonferroni’s multiple comparisons post hoc test. ** *p* < 0.01; *** *p* < 0.001 indicate a significant difference compared with the naïve group. Abbreviations: N—naïve, V—vehicle; MIRO—mirogabalin.

In mice subjected to CCI, on day 7, the mRNA expression levels of microglia/macrophages *Iba-1* [F_(2,24)_ = 56.12, *p* < 0.0001], (Figure 4A) and microglia *Tmem119* [F_(2,24)_ = 13.41, *p* = 0.0001] (Figure 4B) were increased in the spinal cord compared with naïve mice. Similarly, a significant upregulation of astroglial marker *Gfap* [F_(2,23)_ = 0.7325, *p* = 0.4916] (Figure 4C) and lymphocyte T *Cd8* [F_(2,23)_ = 8.400, *p* = 0.0018] (Figure 4E) mRNAs was observed compared with that in naïve mice. Repeated *i.p.* administration of mirogabalin did not affect spinal mRNA levels of *Iba-1, Tmem119*, *Gfap, Cd4* and *Cd8* (Figure 4A–E). No changes were observed in the level of *Cd4* mRNA in the spinal cord in the CCI-exposed vehicle- and mirogabalin-treated mice (Figure 4D).

### 2.5. Effects of Repeated i.p. Administration of Mirogabalin on mRNA Levels of Pro-(IL1-Beta, IL-6, IL-18) and Anti-(IL-1alpha, IL-10, IL-18BP) Nociceptive Factors Measured on Day 7 after CCI in the Spinal Cord in Mice

**Figure 5:**
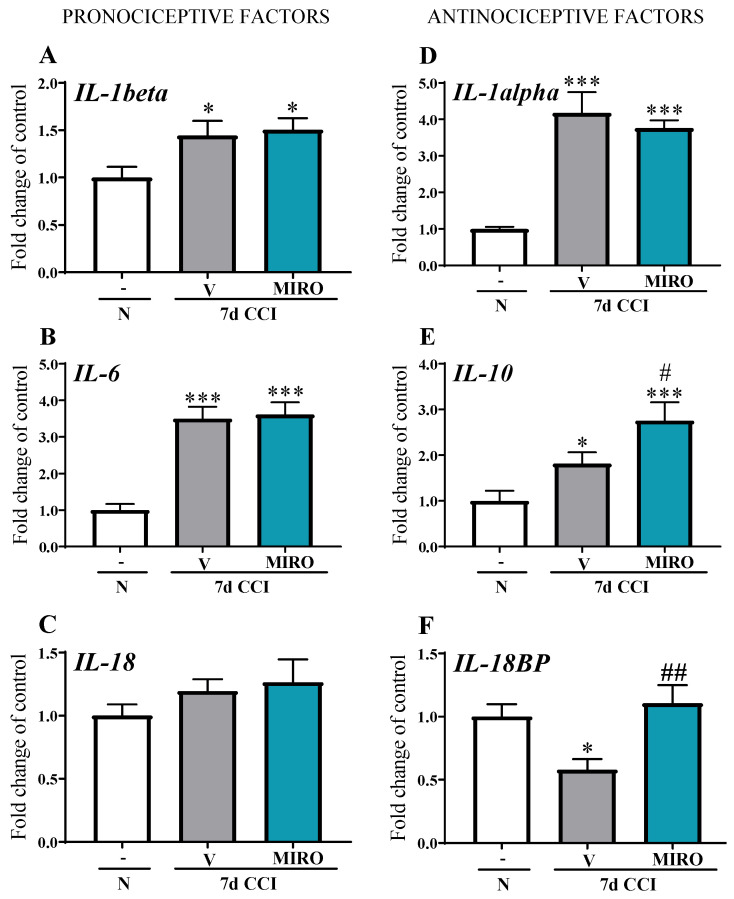
Effect of repeated intraperitoneal (*i.p.*) administration of mirogabalin (MIRO; 20 mg/kg) on *IL-1beta* (**A**), *IL-6*, (**B**), *IL-18*, (**C),** *IL-1alpha* (**D**), *IL-10* (**E**), *IL-18BP* (**F**) mRNA levels in the spinal cord on day 7 post-CCI in mice. The data for the biochemical studies were analysed as fold changes compared with the controls and represent the normalized averages derived from the RT-qPCR threshold cycles of 4–10 samples from each group. Intergroup differences were analysed based on one-way ANOVA with Bonferroni’s multiple comparisons post hoc test. * *p* < 0.05; *** *p* < 0.001 indicate a significant difference compared with the naïve group, # *p* < 0.05, ## *p* < 0.01 indicate differences between the V- and MIRO-treated CCI-exposed mice. Abbreviations: N—naïve, V—vehicle; MIRO—mirogabalin.

In the spinal cord, upregulation of *IL1-beta* mRNA [F_(2,24)_ = 4.201, *p* = 0.0273] and *IL-6* mRNA [F_(2,21)_ = 27.21, *p* < 0.0001] was observed in mice subjected to CCI compared with naïve mice (Figure 5A,B). No changes were observed in the level of *IL-18* mRNA in the spinal cord (Figure 5C). Repeated *i.p.* administration of mirogabalin did not influence the mRNA levels of the pronociceptive factors *IL-1beta, IL-6*, and *IL-18* (Figure 5A–C).

Both *IL-1alpha* and *IL-10* mRNA were upregulated in the spinal cord ([F_(2,21)_ = 16.60, *p* < 0.0001 and F_(2,17)_ = 9.234, *p* = 0.0019], respectively) (Figure 5D,E) after CCI compared with naïve mice. We demonstrated that *IL-18BP* mRNA levels were decreased 7 days after CCI [F_(2,21)_ = 7.127, *p* = 0.0043] compared with that in naïve mice (Figure 5F). Importantly, repeated *i.p.* administration of mirogabalin enhanced the mRNA levels of *IL-10* [F_(2,17)_ = 9.234, *p* = 0.0019] and *IL-18BP* [F_(2,21)_ = 7.127, *p* = 0.0043] in the spinal cord (Figure 5E,F).

### 2.6. Effects of Repeated i.p. Administration of Mirogabalin on Pronociceptive Neuropeptide (Substance P, CGRP, Neuropeptide Y) mRNA Levels Measured on Day 7 after CCI in the Spinal Cord in Mice

**Figure 6:**
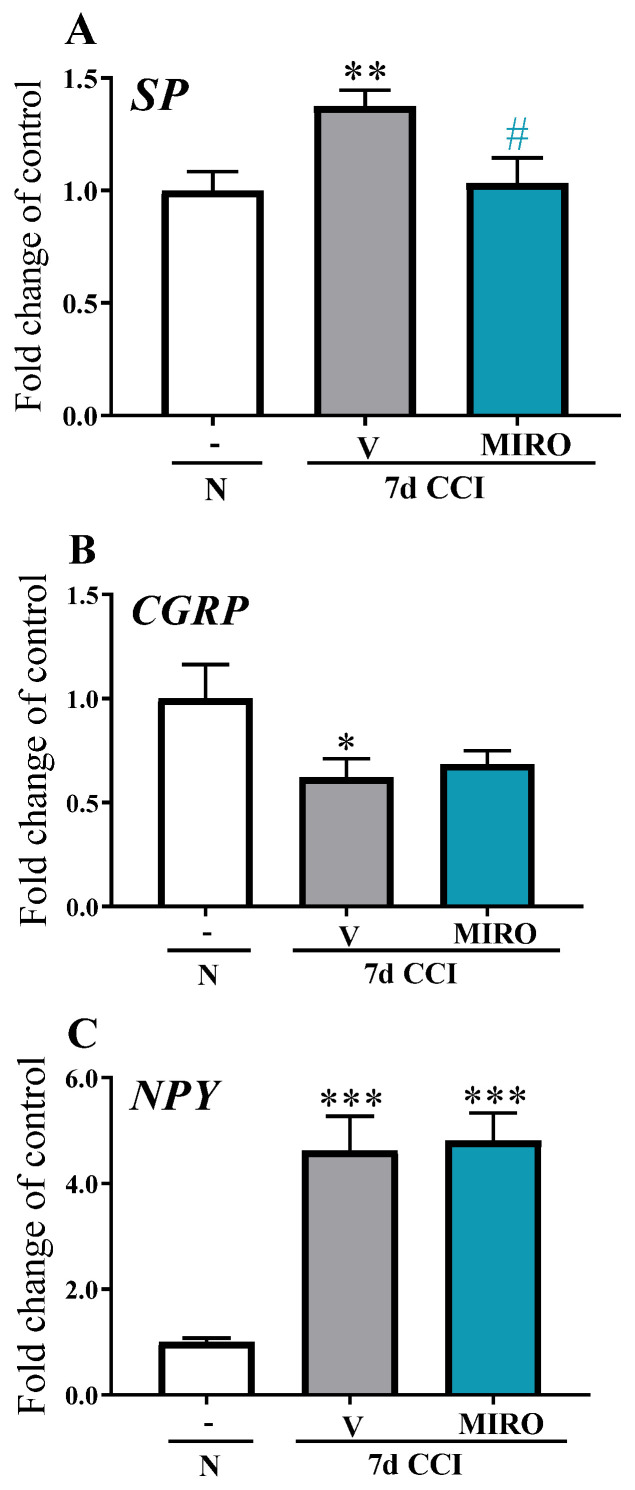
Effect of repeated intraperitoneal (*i.p.*) administration of mirogabalin (MIRO; 20 mg/kg) on *substance P* (**A**), *CGRP* (**B**), and *neuropeptide Y* (**C**) mRNA levels in the spinal cord on day 7 post-CCI in mice. The data for the biochemical studies were analysed as fold changes compared with the controls and represent the normalized averages derived from the RT-qPCR threshold cycles of 6–10 samples from each group. Intergroup differences were analysed based on one-way ANOVA with Bonferroni’s multiple comparisons post hoc test. * *p* < 0.05, ** *p* < 0.01; *** *p* < 0.001 indicate a significant difference compared with the naïve group. # *p* < 0.05 indicates differences between the V- and MIRO-treated CCI-exposed mice. Abbreviations: N—naïve, V—vehicle; MIRO—mirogabalin.

In mice subjected to CCI, *substance P* mRNA expression in the spinal cord was upregulated [F_(2,21)_ = 6.611, *p* = 0.0059] compared with that in naïve mice (Figure 6A). Repeated *i.p.* mirogabalin injections significantly decreased *substance P* mRNA levels compared with vehicle-treated animals. (Figure 6A). Moreover, CCI induced the downregulation of *CGRP* mRNA levels [F_(2,22)_ = 3.223, *p* = 0.0592], although compared to naïve mice, mirogabalin administration did not influence the *CGRP* mRNA levels (Figure 6B). The mRNA level of *neuropeptide Y* was significantly upregulated [F_(2,21)_ = 14.42, *p* = 0.0001] in mice subjected to CCI compared with naïve mice (Figure 6C); however, mirogabalin had no influence on its elevated level.

### 2.7. Effects of Repeated i.p. Mirogabalin Administration on Opioid Prohormone (Pomc, Penk, Pdyn, Pnoc) and Receptor (Mor, Dor, Kor, Nor) mRNA Levels Measured on Day 7 after CCI in the Spinal Cord in Mice

**Figure 7:**
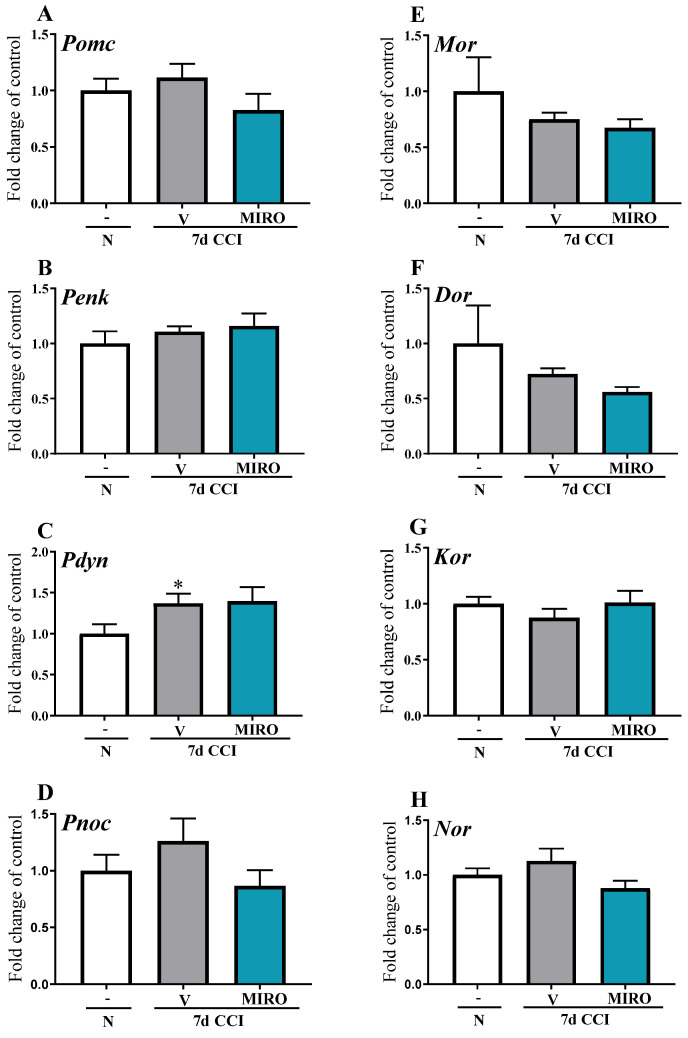
Effect of repeated intraperitoneal (*i.p.*) administration of mirogabalin (MIRO; 20 mg/kg) on the *Pomc* (**A**), *Penk* (**B**), *Pdyn* (**C**), *Pnoc* (**D**) and *Mor* (**E**), *Dor* (**F**), *Kor* (**G**), *Nor* (**H**) mRNA levels in the spinal cord on day 7 post-CCI in mice. The data for biochemical studies were analysed as fold changes compared with the controls and represent normalized averages derived from the RT-qPCR threshold cycles of 5–11 samples from each group. Intergroup differences were analysed based on one-way ANOVA with Bonferroni’s multiple comparisons post hoc test. * *p* < 0.05 indicates a significant difference compared with the naïve group. Abbreviations: N—naïve, V—vehicle; MIRO—mirogabalin.

In the spinal cord, no changes in *Pomc, Penk, Pnoc*, *Mor, Dor, Kor* or *Nor* mRNA levels were observed in either vehicle- or mirogabalin-treated mice or naïve animals (Figure 7A–H). The *Pdyn* mRNA level increased in vehicle-treated mice [F_(2,20)_ = 2.705, *p* = 0.0913] compared with naïve animals (Figure 7C). Repeated *i.p.* administration of mirogabalin did not influence spinal mRNA levels of opioid prohormones *(Pomc, Penk, Pdyn,* and *Pnoc)* or receptors *(Mor, Dor, Kor,* and *Nor).*

## 3. Discussion

In the present study, we demonstrated that single and repeated *i.p.* administration of mirogabalin reduces tactile and thermal hypersensitivity and does not influence motor function in mice subjected to chronic constriction sciatic nerve injury. Similar results were obtained by Murasawa et al. in rats after a single oral mirogabalin administration. The authors showed that mirogabalin diminished both tactile hypersensitivity and anxiety-related behaviour and concluded that mirogabalin may provide effective pain relief and anxiety reduction in patients with neuropathic pain [10]. These results are consistent with our findings. Mirogabalin seems to be effective following peripheral and central nervous system injuries. In 2018, Domon et al. published a study showing that 28 days after spinal cord injury, mirogabalin-treated rats obtained significant pain relief. The authors suggested that mirogabalin may prove to be an effective drug in relieving neuropathic pain in patients with spinal cord injury [11]. The analgesic potential of mirogabalin was also recently confirmed in two experimental models of fibromyalgia, namely, the Sluka model and intermittent cold stress model. In both models, oral mirogabalin administration resulted in dose-dependent, long-lasting (even up to 8 h) pain relief [12].

Although the analgesic properties of mirogabalin have already been confirmed in some experimental studies of pain of varying aetiologies, the neuroimmunological background of its action remains unclear; hence, we chose it as the subject of our research.

According to recent evidence, the activation of microglial/astroglial and immune cells (e.g., macrophages and T CD4 lymphocytes) is critical for the development and maintenance of neuropathic pain [13,14]. In our previously published studies, we demonstrated the strongest spinal upregulation of the studied mRNA glial and immune cell markers on day 7 after CCI [15,16]; therefore, we decided to examine the effect of repeated mirogabalin administration at this time point. Importantly, we observed enhanced mRNA levels of *Iba-1, Tmem119, Gfap* and *Cd4* after CCI; however, mirogabalin treatment did not influence the observed changes. In an attempt to compare the results of our work with others, we conducted a review of the existing literature but found no papers dealing specifically with the effects of mirogabalin administration on the activation of spinal microglial cells and astrocytes. Current research has focused on the activity of gabapentin in this area. Wodarski et al. implemented a streptozotocin-induced diabetic model of neuropathic pain and found that mechanical hypersensitivity observed in rats was associated with increased spinal microglial activation. Gabapentin delivered orally twice a day for 5 days, 4 weeks after streptozotocin injection, attenuated microglial activation but did not affect astroglia [17]. Similar results were obtained in another experimental pain model of chronic myositis induced by an injection of complete Freund adjuvant (CFA) into the right gastrocnemius muscle of rats. In this study, intrathecal gabapentin attenuated microglia but did not cause astrocyte activation in the dorsal horn of the spinal cord [18]. Similarly, in a CFA-induced monoarthritis model, intraperitoneal gabapentin reduced spinal microglial activation, which was assessed using the microglial marker *Iba-1* [19]. Our findings suggest that in the CCI model, mirogabalin produces its analgesic effect mainly through receptors on neuronal cells because mirogabalin administration did not influence the levels of glial or immune cell markers. Similar conclusions have also been reached by other authors studying the effects of pregabalin in an experimental autoimmune encephalomyelitis model. They observed that pregabalin affects neuronal calcium channels and reduces calcium-mediated neuronal cytotoxicity and damage. Moreover, similar to our study, T cells and microglia were unaffected [20].

Taking into account the available literature data showing that gabapentinoids mainly affect nociceptive transmission via receptors located on neurons, we decided to investigate the effects of mirogabalin on the mRNA of three well-known pronociceptive neuropeptides: *CGRP, substance P, and neuropeptide Y*. We found increased mRNA levels of *neuropeptide Y* and *substance P* in the spinal cord 7 days after CCI in mice, in contrast to *CGRP*. Both upregulated neuropeptides are strongly involved in the pathogenesis of neuropathic pain [21,22]. They are released in the spinal dorsal horn by primary sensory afferent neurons, which contributes to their hypersensitivity [21,22]. Repeated treatment with mirogabalin attenuated the increased levels of *substance P*, which is a neuropeptide present in 50% of C-fibers and 20% of Aδ-fibers [23]. It is well documented that hypersensitivity in neuropathy is caused by the release of *substance P* [21]; hence, the reduced *substance P* mRNA level suggests its contribution to the analgesic effects of mirogabalin. Studies evaluating the effects of mirogabalin on *substance P* release in the neuropathic pain model were not observed in the literature. However, Takasusuki et al. found that gabapentin regulates spinal release of *substance P* from small primary afferents in inflammatory pain (in an experimental formalin pain model), which corresponds with our findings [21].

According to published research, the activation of spinal glial and immune cells is the reason for the strongly enhanced expression of various nociceptive factors [13,16]. It leads to the disruption of the balance between pronociceptive factors and antinociceptive factors [16]. The new neuropathic pain treatment strategy relies on stimulating endogenous antinociceptive factors, which is a more physiologic approach than attempts to completely disrupt pronociceptive mechanisms [14,16]. Although mirogabalin treatment did not influence the spinal levels of *Iba-1, Tmem119, Gfap* and *Cd8* cellular markers, it affected some antinociceptive factors.

Previous literature data has emphasized the importance of immunological factors in the analgesic effect of gabapentinoids [13,24,25,26]; hence, these factors were also a subject of our research. Our study provided the first evidence that mirogabalin can influence some nociceptive interleukins. IL-1 family cytokines play an important role in nociceptive transmission [27,28]. *IL-1beta* and *IL-18* exert pronociceptive properties in contrast to *IL-1alpha* and *IL-18BP* [29,30,31]. It was shown that intrathecal administration of *IL-1beta* induces hypersensitivity, in contrast to antinociception evoked by *IL-1alpha* [29]. In the present study, the mRNA levels of pronociceptive *IL-1beta* and antinociceptive *IL-1alpha* significantly increased after CCI, which corresponds with the findings published to date [29]; however, in our experiment, mirogabalin administration did not influence these changes.

The situation is different in the case of *IL-18/IL18BP. IL-18BP* binds to *IL-18* and blocks its activity by preventing its binding to the receptor, making it a natural endogenous inhibitor of this important pronociceptive factor [30]. Our findings revealed the presence but no spinal upregulation of *IL-18,* as measured 7 days after CCI in mice, as well as after mirogabalin administration. The neutralization of *IL-18* with *IL-18BP* potently reduces inflammation and pain [31]. In our study, repeated administration of mirogabalin led to a significant upregulation of *IL-18BP* mRNA, which may be one of the pathways through which mirogabalin exhibits its analgesic properties.

Numerous studies have reported increased spinal levels of pronociceptive *IL-6* following peripheral nerve injury [29,32], which is consistent with our results. However, mirogabalin had no effect on these changes.

The most powerful endogenous regulator of pronociceptive interleukin function in the development of neuropathic pain is *IL-10* [33]. Our studies provide the first evidence that repeated administration of mirogabalin elevates *IL-10* levels in the spinal cord, which may be one of the reasons for mirogabalin’s analgesic properties. Our findings correspond well with the literature showing that gabapentin administration is associated with upregulation of *IL-10* in the spinal cord in neuropathic pain models [24,25,26]. Moreover, the carrageenan-induced paw oedema model in rats showed that pregabalin also increases the levels of antinociceptive *IL-10* [34]. We are the first to report that mirogabalin enhances the mRNA levels of the spinal antinociceptive factors *IL-10* and *IL-18BP*. This may indicate that gabapentinoids are capable of polarizing immune cells, or that neurons secrete these factors and thus inhibit adverse neuroimmune changes. These hypotheses, however, require further in-depth research.

Our studies provided the first evidence that mirogabalin, administered with opioids (morphine, oxycodone, and buprenorphine) and ketamine, induces better antinociception compared to either alone. Gabapentinoids (gabapentin and pregabalin) are the first choice recommended drugs for the treatment of neuropathic pain in humans [1]. Morphine, oxycodone, and buprenorphine are recommended as third-line treatments based on Grades of Recommendation Assessment, Development and Evaluation (GRADE). Ketamine, as an NMDA antagonist, is regarded as a drug with inconclusive recommendations based on the GRADE classification, mainly due to discrepant findings [35]. The main limitation of the recommended pharmacotherapy in humans is its partial effectiveness, as monotherapy often fails to provide sufficient pain relief or has unacceptable side-effects due to the need for high doses. Thus, in clinical practice, patients with neuropathic pain are often treated with combination therapy. In the literature, evidence has been obtained on the use of different combinations of pharmacological agents, as assessed by Chaparro et al. [4], among others. However, due to limited data, combination therapy was not included in clinical recommendations published by Finnerup et al. [35]. More recently, Danish experts using the Delphi consensus process stated that there is good clinical evidence to support combination therapy with opioids and gabapentinoids, which reflects the clinical experiences and observations to date [36]. The statement was based on randomized controlled trials (RCTs) published by Gilron et al. [7] (2400 mg gabapentin plus 60 mg morphine), Hanna et al. [8] (flexible moderate-dose gabapentin plus flexible moderate-dose oxycodone), and Caraceni et al. [37] (fixed-dose oxycodone plus moderate flexible-dose gabapentin [maximum 1800 mg]) in patients suffering from peripheral NP and in expert clinical practice. In a recent review published by Moisset et al. [6], clear clinical recommendations were made concerning combination therapy in neuropathic pain patients. Based on RCTs, French experts found a weak recommendation for combining gabapentinoids with opioids in the treatment of neuropathic pain. Thus, combination therapy is considered a second-line treatment when monotherapy fails or induces unacceptable side-effects [6]. This clinical report matches the findings of our experimental study. The precise mechanism of potentiation of opioid effects by mirogabalin is unclear. Theoretically, mirogabalin and opioids exhibit different analgesic and antinociceptive mechanisms of action [38]; hence, combining them may be beneficial. Several experimental studies and reviews have revealed the complex nature of neuropathic pain and multiple mechanisms involving nociception in the nervous and immune systems [39]. Animal studies suggest that gabapentinoid activity in modulating neuropathic pain is opioid-independent [40]. Naloxone, a µ-opioid receptor antagonist, does not block acute pregabalin action on abdominal constrictions in the lipopolysaccharide (LPS)-induced rectal hypersensitivity model of visceral pain [41], nor does it block acute gabapentin action in the formalin test (an inflammatory pain model) [42]. The findings of a study conducted by Manandhar et al. suggest that the mechanisms of gabapentinoid action responsible for the opioid-sparing effects and potentiation of morphine analgesia are not associated with a direct or allosteric modulation of μ receptor activity [43]. The available data imply that the potential mechanism underlying the effect of gabapentin on opioid analgesia in neuropathic pain involves its beneficial impact on neuroimmune changes. Bao et al. showed that the mechanisms by which gabapentin improves morphine’s antinociceptive effects in neuropathic pain involve the upregulation of antinociceptive *IL-10* expression in the rat spinal cord [26], which is consistent with our findings obtained with mirogabalin administration in a mouse model of neuropathic pain.

In our study, a single administration of mirogabalin combined with ketamine resulted in a much more effective antinociceptive effect in the experimental neuropathic pain model than each of these drugs administered separately. A similar effect was observed clinically after gabapentin and ketamine administration [9]. Amr noted that in patients with neuropathic pain caused by spinal cord injury, a low-dose ketamine infusion administered daily for one week as an adjuvant to gabapentin resulted in significantly better pain relief than gabapentin alone. Interestingly, the analgesic effect of ketamine was maintained for 2 weeks after the infusion was discontinued. The molecular background of the phenomenon in question was recently proposed by Chen et al. These authors suggested a mechanism that involves the formation of complexes between NMDA receptors and α2δ-1 subunits of voltage-gated Ca^2+^ channels. They also showed that α2δ-1 subunits physically interact with NMDA receptors through their C-terminus and form heteromeric complexes with NMDA receptors [44]. In turn, the interactions between α2δ-1 subunits and NMDA receptors activate the surface trafficking of these complexes and increase their synaptic expression in neuropathic pain. Therefore, in our opinion, mirogabalin can reduce neuropathic pain by interacting with α2δ-1 subunit-bound NMDA receptors, which inhibits the synaptic transfer and activity of α2δ-1/NMDA receptor complexes. This mechanism could be one of the reasons behind the observed better analgesic efficacy of combination therapy of mirogabalin with ketamine used in our study. The study by Meymandi et al. supported the role of NMDA receptors on the antinociceptive effect of pregabalin. Authors found that NMDA agonists reduced the antinociceptive effect of pregabalin, unlike MK801, an NMDA antagonist [45]. Importantly, mirogabalin has better selectivity for α2δ-1 and α2δ-2 subunits and slower dissociation rate from the α2δ-1 subunits than from the α2δ-2 subunits of Voltage Gated Calcium Channels compared to gabapentin and pregabalin, which results in its higher analgesic efficacy, better safety profile, and relatively lower incidence of adverse effects compared to gabapentin and pregabalin [3]. Therefore, theoretically, the effects of the combination therapy of mirogabalin with other compounds should be more efficient, compared to the combination therapy of already used gabapentinoids. This assumption requires confirmation in further research.

## 4. Materials and Methods

### 4.1. Animals

Adult male Albino-Swiss CD-1 mice (age 4–5 weeks, weighing 20–25 g), purchased from Charles River Laboratories (Hamburg Germany), were placed in sawdust-lined cages, ten per cage, under controlled conditions (temperature 21 ± 2 °C; 12 h light/dark cycle; light on at 6 a.m.), and provided food and water as needed. All the experiments were performed in accordance with the principles set by the International Society for the Study of Pain [46] and the NIH Guidelines for the Care and Use of Laboratory Animals. The study was approved by the Local Ethics Committee Branch II, National Ethics Committee for Experiments on Animals, Institute of Pharmacology, Polish Academy of Sciences (approval no. 236/2020 Krakow, Poland). Precautions were taken to minimize animal suffering, as well as the number of animals used (3R policy).

### 4.2. Sciatic Nerve Surgery

The chronic constriction injury (CCI) procedure was performed on test mice using isoflurane anesthesia (2% isoflurane in 100% oxygen at 1.5 L min^−1^), as described by Bennett and Xie [47] and modified by Mika et al. [48]. Following an incision below the right hip bone, the sciatic nerve was exposed, and three ligatures (3/0 silk) were made around it distal to the sciatic notch, spaced at 1 mm, until a twitch in the limb was noted. Afterwards, long-lasting thermal and tactile hypersensitivity was observed. Control animals were not subjected to the procedure.

### 4.3. Drug Administration

The authors used the following drugs at the following doses: mirogabalin (MIRO; 1, 10, 20, 40 mg/kg, MedChem Express, Monmouth Junction, NJ, USA), morphine hydrochloride (M; 5 mg/kg; Fagron, Krakow, Poland), buprenorphine (B; 5 mg/kg; Polfa S.A., Warsaw, Poland), oxycodone (O; 5 mg/kg; Norpharma A/S, Denmark), and ketamine (K; 15 mg/kg; Panpharma UK Ltd., Horsham, UK). The scheme of the experiments used in our current study were based on our previous published paper [48]. The dosages of morphine and buprenorphine were chosen based on our own studies [49], and the dosage of oxycodone was chosen based on our own unpublished data and literature [50,51]. The dosage of ketamine was chosen based on the literature [52]. Before intraperitoneal administration (*i.p.*) to test mice, all the drugs were dissolved in water (*aqua pro-injection*). Vehicle (*aqua pro-injection*) was given to the control group according to the same protocol. During the study, no adverse effects of opioids or mirogabalin were noted.

### 4.4. Behavioural Tests

Behavioural tests were conducted between 8 a.m. and 12 p.m. Mirogabalin was administered in two experimental schedules. The effect of single *i.p.* mirogabalin administration at different doses on pain-related behaviour was measured 11 days after CCI. Mirogabalin (20 mg/kg) was also administered 16 h and 1 h before CCI and afterwards twice a day for 7 days. On day 7 after CCI at 1 h after the last administration of the drug, tactile hypersensitivity was assessed using the von Frey test, and thermal hypersensitivity was assessed using the cold plate test. Additionally, on day 7 after CCI, single *i.p.* injection of mirogabalin was performed. Next, vehicle-treated and mirogabalin-treated mice received a single *i.p.* vehicle, morphine (5 mg/kg), buprenorphine (5 mg/kg), oxycodone (5 mg/kg), or ketamine (15 mg/kg) injection 1 h after mirogabalin, and then 30 min later, von Frey and/or cold plate tests were performed.

#### 4.4.1. Von Frey Test

Before the experiment, mice were put for 5 min in plastic cages with wire-mesh floors. Using the von Frey test [48], tactile hypersensitivity to non-noxious stimuli was measured and expressed as pressure [g]. A set of calibrated nylon monofilaments (0.6–6 g; Stoelting) was incrementally applied to the midplantar surface of each mouse’s ipsilateral hind paw until a response consistent with pain behaviour was noted. The latter comprised: rapid paw withdrawal, shaking, and licking. In the von Frey test, the findings are expressed as pressure [g] applied with the filament; the cut-off latency was 6 g.

#### 4.4.2. Cold Plate Test

The results of the cold plate test (Cold/Hot Plate Analgesia Meter, No. 05044, Columbus Instruments, Ugo Basile, Gemonio, Italy) was used as an indicator of the mice’s sensitivity to noxious thermal stimulation as described above [48]. The plate temperature was 2 °C, whereas the cut-off latency was 30 s. After the mice were put on the cold plate, the time until they lifted the injured paw was noted. In CCI-exposed mice, the injured (right) paw always responded first; hence, the treatment effect means the response of the ipsilateral hind paw.

### 4.5. RT-qPCR

Ipsilateral fragments of the dorsal part of the lumbar (L4–L6) spinal cord were removed immediately after decapitation on day 7, following CCI and 4 h after the last mirogabalin administration. The tissue samples were put individually in tubes containing RNAlater (Qiagen Inc., Hilden, Germany) and then stored at −80 °C for RNA isolation. Total RNA extraction was performed using TRIzol reagent (Invitrogen) based on Chomczynski and Sacchi’s protocol [53,54]. RNA concentration was measured by DeNovix DS-11 (Wilmington, NC, USA). Reverse transcription was performed on 1 µg of total RNA using Omniscript reverse transcriptase (Qiagen Inc.) at 37 °C for 60 min. The reverse transcription reaction mixes also contained RNase inhibitor (rRNasin, Promega, Mannheim, Germany) and an oligo-(dT16) primer (Qiagen Inc.). The obtained cDNA was diluted 1:10 with RNase/DNase-free H_2_O, and from each sample, ~50 ng of cDNA was used for each quantitative real-time PCR (RT-qPCR) reaction. RT-qPCR was conducted using Assay-On-Demand TaqMan probes based on the manufacturer’s protocol (Applied Biosystems, Foster City, CA, USA) and run on an iCycler device (Bio–Rad, Hercules, Poland). The following TaqMan primers were used in the study: Mm03024075_m1 (*Hprt*), Mm00479862_g1 (*Iba-1*), Mm00525305_m1 (*Tmem119*), Mm01253033_m1 (*Gfap*), Mm00442754_m1 (*Cd4*), Mm01182107_g1 (*Cd8*), Mm00434228_m1 (*IL-1beta*), Mm00446190_m1 (*IL-6*), Mm00434225_m1 (*IL-18*), Mm00439620_m1 (*IL-1alpha*), Mm01288386_m1 (*IL-10*), Mm00456733_m1 (*IL-18BP*), Mm00436881_m1 (*Substance P, SP*), Mm00801463_g1 (*CGRP*), Mm00445771_m1 (*Neuropeptide Y, NPY*), Mm00435874_m1 (*Pomc*), Mm01212875_m1 (*Penk*), Mm00457573_m1 (*Pdyn*), Mm01314909_m1 (*Pnoc*), Mm01188089_m1 (*Mor*), Mm00443063_m1 (*Dor*), Mm01230885_m1 (*Kor*), Mm00440563_m1 (*Nor*). Cycle threshold values were computed using the default parameters of CFX Manager v.2.1 software. The relative RNA abundance was expressed as 2^-(threshold cycle)^. No significant changes to the *Hprt* gene, used as an endogenous control, were observed across the groups (data not shown).

### 4.6. Data Analysis

The behavioural and biochemical findings are expressed as means ± SEM. On completion of one-way ANOVA, differences between groups were also analysed using Bonferroni’s post hoc test. Additionally, the results were evaluated using two-way ANOVA to determine the time × drug interaction, if applicable (Figure 1). Significant differences in comparisons with naïve animals are indicated by * *p* < 0.05; Significant differences compared with V-treated CCI-exposed mice are indicated by # *p* < 0.05; Significant differences between the MIRO+V- and MIRO+M-treated CCI-exposed mice or MIRO+V, and MIRO+B or MIRO+V- and MIRO+O or MIRO+V- and MIRO+K-treated CCI are indicated by ^ *p* < 0.05. Significant differences between V+M and MIRO+M-treated CCI-exposed mice or V+B and MIRO+B-treated CCI-exposed mice, V+O and MIRO+O-treated CCI-exposed mice or V+K and MIRO+K-treated CCI-exposed mice are indicated by & *p* < 0.05. The analysis and figures were prepared using GraphPad Prism v.9.0 (GraphPad Software, San Diego, CA, USA).

## 5. Conclusions

In summary, we found evidence in our study for the efficacy of mirogabalin in the treatment of neuropathic pain. The drug decreases tactile and thermal hypersensitivity after repeated or even single administrations once neuropathic pain symptoms have fully developed. Our findings suggest that several indirect mechanisms may be responsible for mirogabalin’s analgesic action, not only its interactions with spinal α2δ subunits of calcium channels. We are the first to report that mirogabalin enhances the mRNA expression of spinal antinociceptive factors, such as *IL-10* and *IL-18BP*, and reduces the mRNA expression of pronociceptive *substance P*. Importantly, mirogabalin administered with morphine, buprenorphine, and oxycodone induces better antinociception in a neuropathic pain model, which suggests that it can be used clinically, similar to pregabalin and gabapentin. Moreover, mirogabalin potentiates the antinociceptive effects of ketamine. Our findings support the hypothesis that pharmacological improvement of opioid and ketamine analgesia by mirogabalin administration may represent a new and more effective therapeutic strategy in neuropathic pain treatment. In our view, given the promising clinical data and our findings, mirogabalin is likely to become a drug with a greater therapeutic analgesic effect in neuropathic pain.

## Data Availability

Data is contained within the article.

## References

[1] Finnerup N.B., Haroutounian S., Kamerman P., Baron R., Bennett D.L., Bouhassira D., Cruccu G., Freeman R., Hansson P., Nurmikko T. (2016). Neuropathic pain: An updated grading system for research and clinical practice. Pain.

[2] Daiichi Sankyo Company (2019). Tarlige^®^ Tablets: Prescribing Information. http://www.info.pmda.go.jp/downf.

[3] Zajączkowska R., Mika J., Leppert W., Kocot-Kępska M., Malec-Milewska M., Wordliczek J. (2021). Mirogabalin—A Novel Selective Ligand for the α2δ Calcium Channel Subunit. Pharmaceuticals.

[4] Chaparro L.E., Wiffen P.J., Moore R.A., Gilron I. (2012). Combination pharmacotherapy for the treatment of neuropathic pain in adults. Cochrane Database Syst. Rev..

[5] Madden K., Haider A., Rozman De Moraes A., Naqvi S.M., Enriquez P.A., Wu J., Williams J., Liu D., Bruera E. (2021). Frequency of Concomitant Use of Gabapentinoids and Opioids among Patients with Cancer-Related Pain at an Outpatient Palliative Care Clinic. J. Palliat. Med..

[6] Moisset X., Bouhassira D., Avez Couturier J., Alchaar H., Conradi S., Delmotte M.H., Lanteri-Minet M., Lefaucheur J.P., Mick G., Piano V. (2020). Pharmacological and non-pharmacological treatments for neuropathic pain: Systematic review and French recommendations. Rev. Neurol..

[7] Gilron I., Bailey J.M., Tu D., Holden R.R., Weaver D.F., Houlden R.L. (2005). Morphine, gabapentin, or their combination for neuropathic pain. N. Engl. J. Med..

[8] Hanna M., O’Brien C., Wilson M.C. (2008). Prolonged-release oxycodone enhances the effects of existing gabapentin therapy in painful diabetic neuropathy patients. Eur. J. Pain.

[9] Amr Y.M. (2010). Multi-Day Low Dose Ketamine Infusion as an adjuvant to Oral Gabapentin in Spinal Cord Injury Related Chronic Pain: A Prospective, Randomized, Double Blind Trial. Pain Physician.

[10] Murasawa H., Kobayashi H., Saeki K., Kitano Y. (2020). Anxiolytic effects of the novel α2δ ligand mirogabalin in a rat model of chronic constriction injury, an experimental model of neuropathic pain. Psychopharmacology.

[11] Domon Y., Kitano Y., Makino M. (2018). Analgesic effects of the novel α2δ ligand mirogabalin in a rat model of spinal cord injury. Pharmazie.

[12] Saeki K., Yasuda S.I., Kato M., Kano M., Domon Y., Arakawa N., Kitano Y. (2019). Analgesic effects of mirogabalin, a novel ligand for α2δ subunit of voltage-gated calcium channels, in experimental animal models of fibromyalgia. Naunyn Schmiedebergs Arch. Pharm..

[13] Austin P.J., Moalem-Taylor G. (2010). The neuro-immune balance in neuropathic pain: Involvement of inflammatory immune cells, immune-like glial cells and cytokines. J. Neuroimmunol..

[14] Mika J., Zychowska M., Popiolek-Barczyk K., Rojewska E., Przewlocka B. (2013). Importance of glial activation in neuropathic pain. Eur. J. Pharmacol..

[15] Mika J., Osikowicz M., Rojewska E., Korostynski M., Wawrzczak-Bargiela A., Przewlocki R., Przewlocka B. (2009). Differential activation of spinal microglial and astroglial cells in a mouse model of peripheral neuropathic pain. Eur. J. Pharmacol..

[16] Rojewska E., Popiolek-Barczyk K., Jurga A.M., Makuch W., Przewlocka B., Mika J. (2014). Involvement of pro- and antinociceptive factors in minocycline analgesia in rat neuropathic pain model. J. Neuroimmunol..

[17] Wodarski R., Clark A.K., Grist J., Marchand F., Malcangio M. (2009). Gabapentin reverses microglial activation in the spinal cord of streptozotocin-induced diabetic rats. Eur. J. Pain.

[18] Rosa A.S., Freitas M.F., Rocha I.R.C., Chacur M. (2017). Gabapentin decreases microglial cells and reverses bilateral hyperalgesia and allodynia in rats with chronic myositis. Eur. J. Pharmacol..

[19] Yang J.L., Xu B., Li S.S., Zhang W.S., Xu H., Deng X.M., Zhang Y.O. (2012). Gabapentin reduces CX3CL1 signaling and blocks spinal microglial activation in monoarthritic rats. Mol. Brain.

[20] Hundehege P., Fernandez-Orth J., Römer P., Ruck T., Müntefering T., Eichler S., Cerina M., Epping L., Albrechta S., Menke A.F. (2018). Targeting Voltage-Dependent Calcium Channels with Pregabalin Exerts a Direct Neuroprotective Effect in an Animal Model of Multiple Sclerosis. Neurosignals.

[21] Takasusuki T. (2011). The Effects of Intrathecal and Systemic Gabapentin on Spinal Substance P Release. Anesth. Analg..

[22] Fu W., Wessel C.R., Taylor B.K. (2020). Neuropeptide Y tonically inhibits an NMDAR → AC1 → TRPA1/TRPV1 mechanism of the affective dimension of chronic neuropathic pain. Neuropeptides.

[23] McCarthy P.W., Lawson S.N. (1989). Cell type and conduction velocity of rat primary sensory neurons with substance P-like immunoreactivity. Neuroscience.

[24] Dias J.M., de Brito T.V., de Aguiar Magalhaes D. (2014). Gabapentin, a synthetic analogue of gamma aminobutyric acid, reverses systemic acute inflammation and oxidative stress in mice. Inflammation.

[25] Lee B.S., Jun G., Kim S. (2013). Intrathecal gabapentin increases interleukin-10 expression and inhibits proinflammatory cytokine in a rat model of neuropathic pain. J. Korean Med. Sci..

[26] Bao Y.H., Zhou Q.H., Chen R. (2014). Gabapentin enhances the morphine antinociceptive effect in neuropathic pain via the interleukin-10-heme oxygenase-1 signalling pathway in rats. J. Mol. Neurosci..

[27] Malcangio M., Bowery N.G., Flower R.J., Perretti M. (1996). Effect of interleukin-1 beta on the release of substance P from rat isolated spinal cord. Eur. J. Pharmacol..

[28] Kawasaki Y., Zhang L., Cheng J.K., Ji R.R. (2008). Cytokine mechanisms of central sensitization: Distinct and overlapping role of interleukin-1beta, interleukin-6, and tumor necrosis factor-alpha in regulating synaptic and neuronal activity in the superficial spinal cord. J. Neurosci..

[29] Mika J., Korostynski M., Kaminska D., Wawrzczak-Bargiela A., Osikowicz M., Makuch W., Przewlocki R., Przewlocka B. (2008). Interleukin-1 alpha has antiallodynic and antihyperalgesic activities in a rat neuropathic pain model. Pain.

[30] Dinarello C.A., Fantuzzi G. (2003). Interleukin-18 and host defense against infection. J. Infect. Dis..

[31] Pilat D., Piotrowska A., Rojewska E., Jurga A., Ślusarczyk J., Makuch W., Basta-Kaim A., Przewlocka B., Mika J. (2016). Blockade of IL-18 signaling diminished neuropathic pain and enhanced the efficacy of morphine and buprenorphine. Mol. Cell Neurosci..

[32] Dubový P., Brázda V., Klusáková I., Hradilová-Svíženská I. (2013). Bilateral elevation of interleukin-6 protein and mRNA in both lumbar and cervical dorsal root ganglia following unilateral chronic compression injury of the sciatic nerve. J. Neuroinflamm..

[33] Ledeboer A., Jekich B., Sloane E.M., Mahoney J.F., Langer S.J., Milligan E.D., Martin D., Maier S.F., Johnson K.W., Leinwand L.A. (2007). Intrathecal Interleukin-10 Gene Therapy Attenuates Paclitaxel-Induced Mechanical Allodynia and Proinflammatory Cytokine Expression in Dorsal Root Ganglia in Rats. Brain Behav. Immun..

[34] Kilic F.S., Kaygisiz B., Aydin S., Yildirim C., Karimkhani H., Oner S. (2018). Pregabalin attenuates carrageenan-induced acute inflammation in rats by inhibiting proinflammatory cytokine levels. Eurasian J. Med..

[35] Finnerup N.B., Attal N., Haroutounian S., McNicol E., Baron R., Dworkin R.H., Gilron I., Haanpää M., Hansson P., Jensen T.S. (2015). Pharmacotherapy for neuropathic pain in adults: A systematic review and meta-analysis. Lancet Neurol..

[36] Holbech J.V., Jung A., Jonsson T., Wanning M., Bredahl C., Bach F.W. (2017). Combination treatment of neuropathic pain: Danish expert recommendations based on a Delphi process. J. Pain Res..

[37] Caraceni A., Zecca E., Bonezzi C., Arcuri E., Tur R.Y., Maltoni M., Visentin M., Gorni G., Martini C., Tirelli W. (2004). Gabapentin for neuropathic cancer pain: A randomized controlled trial from the Gabapentin Cancer Pain Study Group. J. Clin. Oncol..

[38] Stein C. (2018). New concepts in opioid analgesia. Expert Opin. Investig. Drugs.

[39] Colloca L., Ludman T., Bouhassira D., Baron R., Dickenson A.H., Yarnitsky D., Freeman R., Truini A., Attal N., Finnerup N.B. (2017). Neuropathic pain. Nat. Rev. Dis. Primers.

[40] Kremer M., Yalcin I., Nexon L., Wurtz X., Ceredig R.A., Daniel D., Hawkes R.A., Salvat E., Barrot M. (2016). The antiallodynic action of pregabalin in neuropathic pain is independent from the opioid system. Mol. Pain.

[41] Eutamene H., Coelho A.M., Theodorou V., Toulouse M., Chovet M., Doherty A., Fioramonti J., Bueno L. (2000). Antinociceptive effect of pregabalin in septic shock induced rectal hypersensitivity in rats. J. Pharmacol. Exp. Ther..

[42] Field M.J., Oles R.J., Lewis A.S., McCleary S., Hughes J., Singhet L. (1997). Gabapentin (neurontin) and S-(ţ)-3-isobutylgaba represent a novel class of selective antihyperalgesic agents. Br. J. Pharmacol..

[43] Manandhar P., Murnion B.P., Grimsey N.L., Connor M., Santiago M. (2021). Do gabapentin or pregabalin directly modulate the μ receptor?. PeerJ.

[44] Chen J., Li L., Chen S., Chen H., Xie J., Sirrieh R.E., MacLean D.M., Zhang Y., Zhou M.H., Jayaraman V. (2018). The α2δ-1-NMDA Receptor Complex Is Critically Involved in Neuropathic Pain Development and Gabapentin Therapeutic Actions. Cell Rep..

[45] Meymandi M.-S., Keyhanfar F., Yazdanpanah O., Heravi G. (2015). The Role of NMDARs Ligands on Antinociceptive Effects of Pregabalin in the Tail Flick Test. Anesth. Pain Med..

[46] Zimmermann M. (1983). Ethical guidelines for investigations of experimental pain in conscious animals. Pain.

[47] Bennett G.J., Xie Y.K. (1988). A peripheral mononeuropathy in rat that produces disorders of pain sensation like those seen in man. Pain.

[48] Mika J., Osikowicz M., Makuch W., Przewlocka B. (2007). Minocycline and pentoxifylline attenuate allodynia and hyperalgesia and potentiate the effects of morphine in rat and mouse models of neuropathic pain. Eur. J. Pharmacol..

[49] Bogacka J., Ciapala K., Pawlik K., Kwiatkowski K., Dobrogowski J., Przeklasa-Muszyńska A., Mika J. (2020). CCR4 antagonist (C021) administration diminishes hypersensitivity and enhances the analgesic potency of morphine and buprenorphine in mouse model of neuropathic pain. Front. Immunol..

[50] Nozaki C., Saitoh A., Tamura N., Kamei J. (2005). Antinociceptive effect of oxycodone in diabetic mice. Eur. J. Pharmacol..

[51] Yang P.P., Yeh G.C., Huang E.Y., Law P.Y., Loh H.H., Tao P.L. (2015). Effects of dextromethorphan and oxycodone on treatment of neuropathic pain in mice. J. Biomed. Sci..

[52] Onaolapo O.J., Paul T.B., Onaolapo A.Y. (2017). Comparative effects of sertraline, haloperidol or olanzapine treatments on ketamine-induced changes in mouse behaviours. Metab. Brain Dis..

[53] Chomczynski P., Sacchi N. (1987). Single-step method of RNA isolation by acid guanidinium thiocyanate-phenol-chloroform extraction. Anal. Biochem..

[54] Chomczynski P., Sacchi N. (2006). The single-step method of RNA isolation by acid guanidinium thiocyanate-phenol-chloroform extraction: Twenty-something years on. Nat. Protoc..

